# Some Distinct Attributes of ZnO Nanorods Arrays: Effects of Varying Hydrothermal Growth Time

**DOI:** 10.3390/ma15175827

**Published:** 2022-08-24

**Authors:** Mohammed Rashid Almamari, Naser M. Ahmed, Araa Mebdir Holi, F. K. Yam, Htet Htet Kyaw, M. A. Almessiere, Mohammed Z. Al-Abri

**Affiliations:** 1 Nanotechnology Research Center, Sultan Qaboos University, P.O. Box 17, Al Khoud, Muscat 123, Oman; 2 School of Physics, Universiti Sains Malaysia, Penang 11800, Malaysia; 3 Research Center, The University of Mashreq, Baghdad 10021, Iraq; 4 Department of Physics, College of Education, University of Al-Qadisiyah, Al-Diwaniyah 58002, Al-Qadisiyah, Iraq; 5 Department of Physics, College of Science, Imam Abdulrahman Bin Faisal University, P.O. Box 1982, Dammam 31441, Saudi Arabia; 6 Department of Biophysics, Institute for Research & Medical Consultatuins (IRMC), Imam Abdulrahman Bin Faisal University, P.O. Box 1982, Dammam 31441, Saudi Arabia; 7 Department of Petroleum and Chemical Engineering, College of Engineering, Sultan Qaboos University, P.O. Box 33, Al Khould, Muscat 123, Oman

**Keywords:** PEC, photoelectrode, photoconversion efficiency, hydrothermal growth time, ZNRAs

## Abstract

This study investigates the growth time effect on the structural, morphological, optical, and photoelectrochemical characteristics of highly oriented ZnO nanorod arrays (ZNRAs). The nanorod arrays were grown on ITO substrates using the unified sol-gel spin coating and hydrothermal techniques. ZnO nanoparticles (ZNPs) were synthesized using the sol-gel spin coating method. In contrast, the hydrothermal method was used to grow the ZnO nanorods. The hydrothermal growth time investigated was between 4 and 12 h. The synthesized ZNRAs were used as the photoanode electrodes to investigate their photoelectrochemical (PEC) electrode potency. The as-prepared ZNRAs were characterized using various analytical tools to determine their structures, morphologies, optical, and photoelectrochemical traits. EDX spectra showed the presence of uncontaminated ZnO chemical composition, and FTIR spectra displayed the various functional groups in the samples. A rod-shaped ZnO nanocrystallite with mean lengths and diameters of 300–500 nm and 40–90 nm, respectively, is depicted. HRTEM images indicated the nucleation and growth of ZNRAs with a lattice fringe spacing of 0.26 nm and a growth lattice planer orientation of [002]. The optimum ZNRAs (grown at 8 h) as photoelectrode achieved a photoconversion efficiency of 0.46% and photocurrent density of 0.63 mA/cm^2^, that was 17 times higher than the one shown by ZNPs with Ag/AgCl as the reference electrode. Both values were higher than those reported in the literature, indicating the prospect of these ZNRAs for photoelectrode applications.

## 1. Introduction

Exploring and developing alternative energy sources became inevitable to combat global warming and climate change due to greenhouse gas emissions. Intensive studies have been performed to create photoelectrochemical cells (PECs) that combine high-performing materials and cost-effective fabrication processes to meet the spike in energy demand in a sustainable manner. It is known that the material qualities alter based on their crystallite sizes and thin film thicknesses. Therefore, nanocrystalline materials electrodes have ushered in a new era in the field of PECs application. The electric and light absorption properties of PECs are potentially determined by the quantum size effects, which are beneficial for varied applications. The efficiency of PECs is determined by the ability to harvest visible light, architectures, charge carriers transport, charge carriers separation, and characteristics of the photoanode materials.

In the basic architecture of a PEC cell, some electrolyte, a working photoelectrode made of semiconducting material (n- or p-type), a counter electrode made of metallic element (preferably platinum, Pt), and a reference electrode are used. The purpose of the reference electrode in the PEC is to determine the half-cell reaction mechanism. During the photoelectrolytic reaction in the electrolyte, many physicochemical processes occur at the semiconductor and semiconductor/electrolyte interfaces. These processes include charge segregations, efficient light absorptions, migration of charge carriers, recombination of charge carriers, and surface redox reactions. In addition, upon illumination, a photoelectrode with an energy band gap lower than the incident photon shows electronic absorption. The valence state electrons are excited to the conduction state, leaving holes in the valence state behind. These mobile electrons in the conduction state and holes in the valence state move through the photoelectrode (semiconductor)/electrolyte interface, undergoing a redox reaction and thus converting the solar energy into chemical energy [1,2,3].

For the PECs electrode fabrication, semiconductors are widely considered one of the most significant materials. ZnO has a comparatively wide band gap semiconductor (band gap energy ranges from 3.2 to 3.4 eV) among other metal-oxide semiconductors [4,5,6] such as TiO_2_ [7], Fe_2_O_3_ [8], and WO_3_ [9]. ZnO has many distinct traits: high breakdown voltage, broad range electric field conditions, good temperature stability, and lower electronic noise [10]. Compared to TiO_2_, ZnO has a 10- to 100-fold higher electron mobility, resulting in improved electron transmission efficiency [11]. Furthermore, ZnO has very high exciton binding energy (60 meV) at room temperature, indicating its prospect for numerous optoelectronic applications [12,13]. Commonly, ZnO nanostructures (ZNSs) show excellent optical properties due to the quantum confinement effect, which is exploited by the photonic and photoelectrical devices [10]. The structure, and electrical and optical characteristics of ZnO have fascinated the gamut of research activities in nanotechnology. Amongst numerous wide energy band gap semiconductors, ZnO exhibits a very high electronic mobility (bulk: 205–300 cm^2^ V s^−1^ and single nanowires: 1000 cm^2^ V s^−1^) and reduced carriers’ recombination loss, which is highly desirable for the PEC applications. Despite many dedicated efforts, ZNSs-based efficient photoelectrodes for PECs remain deficient.

Over the years, several techniques have been developed to produce good quality ZNSs. ZnO nanorod arrays (ZNRAs) were synthesized as arranged thin films on planar surfaces using seeded methods such as electrochemical deposition and hydrothermal approach [14] or seedless methods such as Van der Waals epitaxy [10]. These methods are used to enhance the arrays′ photoelectrochemical efficiency. This approach enables the deposition of ZNRAs arrays on a wide range of substrates, including metals, graphene, and transparent conductive oxides. Techniques such as physical evaporation [15], radio frequency [16], magnetron sputtering [17], electrophoretic deposition [18], and hydrothermal technique [19,20,21] have been utilized to make highly oriented ZNRAs. Consequently, it became essential to develop a simple, low-temperature approach that meets the demands of high-performance PECs while avoiding the use of sophisticated equipment and enabling high precision of growth parameters.

In this study, the hydrothermal approach was chosen to produce ZNRAs because it offers several advantages including low cost, minimal need for sophisticated instrumentation, and the flexibility to deposit a variety of materials on various substrates. These benefits are well-suited for large-scale deposition to vary thin-film characteristics by modifying and manipulating the experimental conditions of the deposition [11]. It can also produce high-quality semiconductor thin films due to the slow growth processes, which allow for better crystallite orientation with improved grain structure and configuration growth [22]. Different lengths and diameters of ZNRAs result from changes in hydrothermal factors such as hydrothermal growth time, affecting the morphological features, crystalline quality, band gap energy, and photoconversion efficiency (*η*%) of PECs. Based on these factors, we used the combined sol-gel spin coating (for producing ZNPs) and hydrothermal method for synthesizing vertically aligned ZNRAs while varying the growth times to enhance the *η*%. Samples were characterized thoroughly using diverse analytical tools to determine their structures, morphologies, optical and photoelectrochemical traits. Both photocurrent density and *η*% improved with increased growth times from 4 to 12 h.

## 2. Materials and Methods

### 2.1. Materials

Pure chemical reagents of zinc acetate dehydrate, [(CH_3_COO)_2_ Zn.2H_2_O] (BDH, Poole, UK), zinc nitrate hexahydrate, [Zn(NO_3_)_2_.6H_2_O] (Sigma, Ronkonkoma, NY, USA), hexamethylenetetramine [C_6_H_12_N_4_] (HMTA, Merck, Darmstadt, Germany), absolute ethanol, and acetone (Merck, Darmstadt, Germany) were purchased and utilized without any refinement. Deionized double distilled water of 18.2 MΩ (Millipore Alpha Q deionized water (DIW) system) was employed in all experimentations.

### 2.2. Preparation of ZNPs Using Spin Coating Method

**Figure 1:**
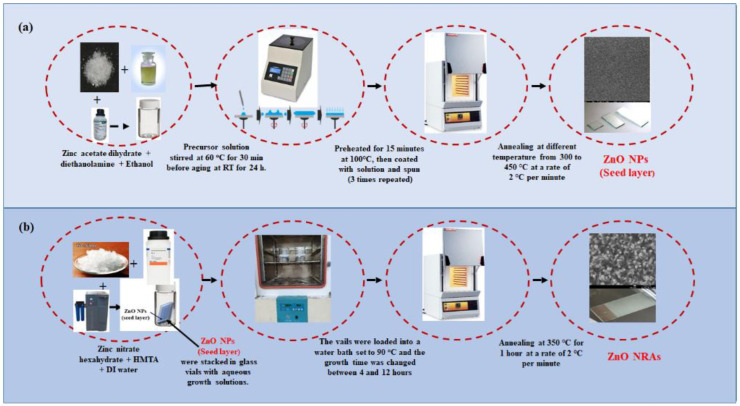
Schematic diagram of (**a**) the fabrication of a ZnO NPs seed layer using sol-gel spin coating and (**b**) the hydrothermal method of synthesized ZnO NRAs.

As illustrated in Figure 1a, indium tin oxide (ITO) glass substrates (resistivity of 10 Ω cm^−2^) were ultrasonically rinsed successively using acetone, 2-propanol, and deionized water each for 15 min at 25 °C. Then, these substrates were thoroughly cleaned again using DIW to remove impurities and then dried with air, activating the substrate surface for ZNSs growth. Unified sol-gel spin coating and hydrothermal methods were used in two steps to make ZNRAs. First, sol-gel spin coating method was used to deposit high density and homogeneous ZNPs seed layers on the substrate’s active surface. In this process, (CH_3_COO)_2_ Zn·2H_2_O (0.1 M) was mixed with ethanol (10 mL) and diethanolamine [CH_2_(OH)CH_2_)]_2_NH of 0.1 M to grow the ZNPs seed layers. To achieve a uniform and stable colloidal suspension, the resultant mixture was whisked at 60 °C for 30 min before being left for about 12 h. Approximately 100 µL of the obtained colloidal suspension was applied on the substrate surface over an area of (1.0 cm × 1.5 cm) and revolved for 40 s at the speed of 3000 rpm to obtain a coating on the ITO surface which were then pre-heated at 100 °C for 15 min. The coating and pre-heating procedure was conducted thrice. Finally, the coated substrates were annealed for 1 h at 400 °C with 2 °C/min rate [23].

### 2.3. Preparation of Vertically Aligned ZNRAs Using Hydrothermal Method

As illustrated in Figure 1b, the hydrothermal method was used to grow ZNRAs wherein a water bath system at 90 °C allowed in controlling growth times between 4 and 12 h. In this process, the ZNPs seed layer-coated substrate was immersed in a sealed glass pot enclosing a mixture of zinc nitrate hexahydrate (40 mM) and HMTA (40 mM) and put it in the oven (Carbolite, Hope, UK) at 90 °C for various growth times to get vertically aligned ZNRAs. Then, the resultant products were cleaned using DIW and air-dried. Finally, ITO substrates coated with ZNRAs were annealed at 350 °C in an electrical furnace (Carbolite CWF 1200, Hope, UK) for 1 h at a heating rate of 2 °C/min. The obtained samples were coded as ZNRAs-4h, ZNRAs-6h, ZNRAs-8h, ZNRAs-10h, and ZNRAs-12h.

### 2.4. Structural, Morphological, and Optical Characterizations of ZNRAs

The physical and chemical properties of the deposited ZNRAs were measured using diverse analytical tools. An ultraviolet-visible spectrophotometer (Perkin Elmer, Lambda 25, Waltham, MA, USA) in the range of 190–1100 nm was used determine the absorption properties of ZNRAs. The morphologies of the samples were studied using the field emission scanning electron microscope (FE-SEM, JSM-7800F, JEOL, Tokyo, Japan). An X-ray diffractometer (Miniflex 600, Rigaku, Tokyo, Japan) was used to determine the crystal structures of the samples, wherein the scanning angle (2θ) range was 20° to 80° at a resolution of 0.02°/s. A Fourier transform infrared (FTIR) spectroscope (PerkinElmer, SpectraOne, Waltham, MA, USA) in the range of 400 to 4000 cm^−1^ (40 scans at a resolution of 4 cm^−1^) was used to identify the chemical functional groups in the samples wherein the attenuated total reflection (ATR) mode was used. The photoluminescence (PL) emission spectra of the ZNRAs were recorded by a fluorescence spectrophotometer (LS 55, Perkin Elmer, Waltham, MA, USA) which used the excitation of 325 nm. The surface composition and chemical state of the ZNRAs were obtained using an X-ray photoelectron spectrometer (XPS, Scienta Omicron, Taunusstein, Germany) that used monochromatic Al-Kα radiation source (1486.6 eV).

### 2.5. Photoelectrochemical Characterizations of ZNRAs

A 3-electrodes PEC was designed with ZNRAs as working photoanode to determine its performance wherein a mixture of Na_2_SO_3_ (0.1 M) and Na_2_S of 0.1 M (pH of 13), Pt wire, and Ag/AgCl served as the electrolyte, counter, and reference electrodes, respectively. A linear sweep voltammeter (LSV, Gamry Instrument framework interface with 1000 E Potentiostat/ Galvanostat/ZRA) was used to measure the photocurrent density (*J_P_* = *J_L_* – *J_D_* in mAcm^−2^) of the PEC. The data was recorded over an applied potential (*V_app_*) range of −1.2 to +1.2 V against Ag/AgCl at the scanning rate of 20 mVs^−1^ with a solar simulator (power of 1.6 kW). A xenon lamp from the Newport system that was equipped with the filter of AM 1.5 G was used for simulating the atmospheric and terrestrial states of the solar irradiation. The intensity of the lamp output was matched with the solar irradiance spectra following the standard of ASTM G173-03(2012). The light from the xenon lamp was focused on the quartz reaction cell over an area of 1 cm × 1 cm of the working electrode, these were placed at a separation of 15 cm. An irradiance of 100 mW/cm^2^ equivalent to one sun lighting was used wherein the manual chopping was utilized for the light source at regular time. The entire PEC measurements were conducted at ambient atmospheric conditions and repeated for 3 times to guarantee the reproducibility of results with statistical accuracy. The values of *η*% of the proposed ZNRAs prepared at various hydrothermal growth times were estimated via [23]:

(1) $$\eta =\frac{J_{ph}\left(1.23-V_{app}\right)}{P_{in}}\times 100\%$$

where *P_in_* is the incident light irradiance (mWcm^−2^), *V_app_* is the external applied voltage, and 1.23 is the standard redox potential of water electrolysis.

## 3. Results and Discussion

### 3.1. Structures and Morphologies of ZNRAs

**Figure 2:**
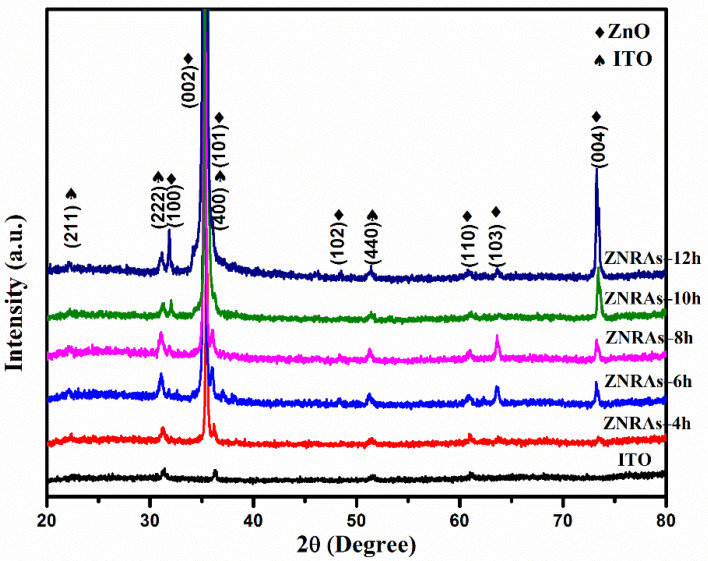
XRD profiles of ITO and ZNRAs made at various hydrothermal growth times.

Figure 2 displays XRD profiles of the ZNPs and ZNRAs synthesized at various growth times from 4 to 12 h. The observed sharp diffraction peaks were enumerated to the wurtzite hexagonal crystal structure of ZnO with lattice parameters a = 3.24 Å and c = 5.20 Å, confirming the JCPDS card No. 04-008-7114. These Bragg’s peaks at 2θ = 31.78°, 34.44°, 36.27°, 47.56°, 60.87°, 63.57°, and 72.61° corresponded to the crystalline lattice planes of (100), (002), (101), (102), (110), (103), and (004). With the increase of growth times, the intensity of the prominent XRD peak at 2θ = 34.4° was enhanced, indicating the preferential growth orientation of ZNRAs lattice plane (002) due to its lower surface free energy along this direction [24,25]. Furthermore, the XRD peaks at 22.06°, 31.32°, 36.30°, and 51.49° were due to the Bragg’s reflection from the ZNRAs lattice planes of (211), (222), (400), and (440), respectively, confirming the cubic structure (JCPDS card No. 01-089-4598). The complete absence of any XRD peak in the proposed ZNRAs clearly indicated their high purity. The mean crystallite sizes (*D*) of ZNRAs were estimated from the full-width at half maximum (FWHM = β) of the intense diffraction peak at 2θ = 34.4° corresponding to the (002) lattice plane wherein the Debye–Scherrer equation was used [26]:

(2) $$D=\frac{k\lambda }{\beta cos\theta }$$

where *λ* = 0.154056 nm is the X-ray wavelength (Cu-Kα radiation) [23,25,27]. The relative intensities of the Bragg peaks of ZNRAs were consistent with the strong growth orientation effects [25]. Irrespective of the growth time, the value of *D* was estimated to be 21.95 ± 2.03 nm, indicating an excellent nanocrystallinity of ZNRAs along the crystallographic c-axis. The intensity of the dominant (002) plane was mainly due to the quantitative concentration of Zn^++^ in the pioneer solution, boosting or inhibiting the growth process of the ZNRAs [20]. It is important to note that ZnO being a polar crystal its positive and negative polar planes are correspondingly enriched in Zn and O [28], wherein the Ostwald ripening is the dominant growth mechanism for the ZnO single crystals [29]. In this process, the larger crystals are grown spontaneously at the expense of smaller ones due to favorable free energy kinetics and thermodynamic stability. Consequently, in the supersaturated media the nucleation of the small crystallites was favored over the bigger ones due to free energy kinetics, wherein the Gibbs–Thomson free energy difference (called higher solubility law), among the larger and tinier ZnO crystallites, played the key role [30]. In addition, the occurrences of the polar and non-polar lattice planes enabled an anisotropic nucleation and growth processes of the nanocrystallites, leading to 1D (nanowires) ZNSs called ZNRs that offered wide surface area to volume ratio effectively [27,31].

**Figure 3:**
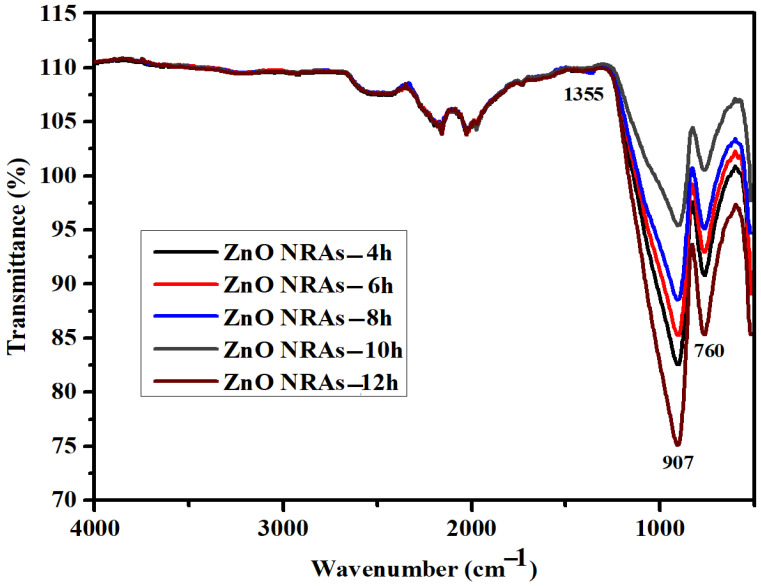
FTIR spectrum of ZNRAs prepared at various hydrothermal growth times.

Figure 3 displays FTIR spectra of the ZNRAs. The observed asymmetric and broad band at 1355 cm^−1^ was due to the hydroxyl (O–H) bonds stretching vibration. In all samples, the bands at 906.7 cm^−1^ and 758.6 cm^−1^, corresponding to the Zn–O bonds vibration [32,33].

**Figure 4:**
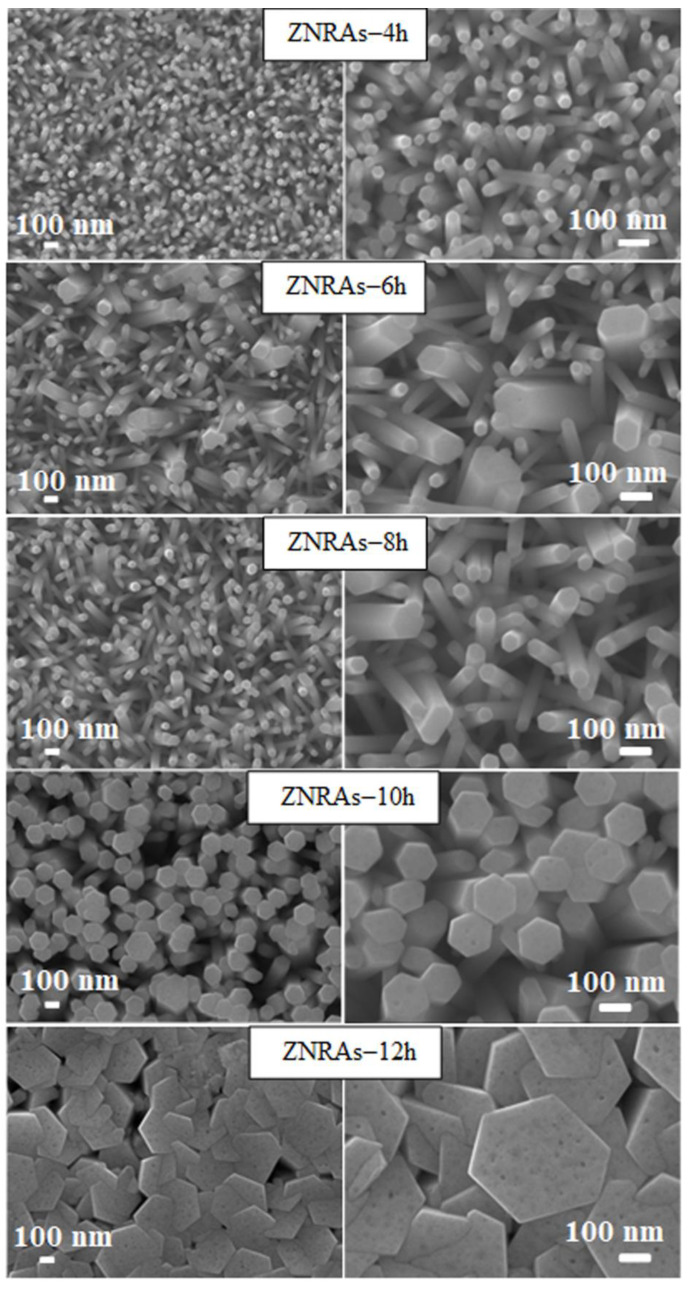
FE-SEM images (top view) of ZNRAs prepared at various hydrothermal growth times.

Figure 4 displays the FE-SEM images (top view) of ZNRAs prepared at various hydrothermal growth times, which showed remarkable effects on the surface morphologies and microstructures of the synthesized ZNRAs. The hexagonal wurtzite nanocrystallites density, side distribution, homogeneity, and uniformity of the ZNRAs were improved significantly with the prolongation of the hydrothermal growth times. ImageJ software was used to estimate the diameters of the ZNRs from the top view FE-SEM images. These diameters were obtained by measuring multiple NRs at various locations and averaging their overall sizes. The mean diameters of the ZNRs synthesized at growth times of 4, 6, 8, 10, and 12 h were 37.16, 65.37, 55.29, 139.15, and 411.79 nm, respectively. Generally, the diameters of the ZNRs increased (except at a growth time of 8 h) with the increase in the hydrothermal growth times. The NRs elongated with the increase of growth times wherein the adjacent thinner NRs merged to form thicker NRs (larger diameters). The merging of the neighboring thinner NRs and formation of the thicker NRs can be ascribed to the shortening of their separation and subsequent attachment of their crystalline lattice planes at longer hydrothermal duration [34,35]. The observed slight decrease in the ZNRs mean diameter at 8 h was mainly due to relatively low contents of Zn^2+^ and OH^−^ in the ZNRAs growth media and ZNRAs dissolution aroused from the reversal of the chemical reaction [23].

**Figure 5:**
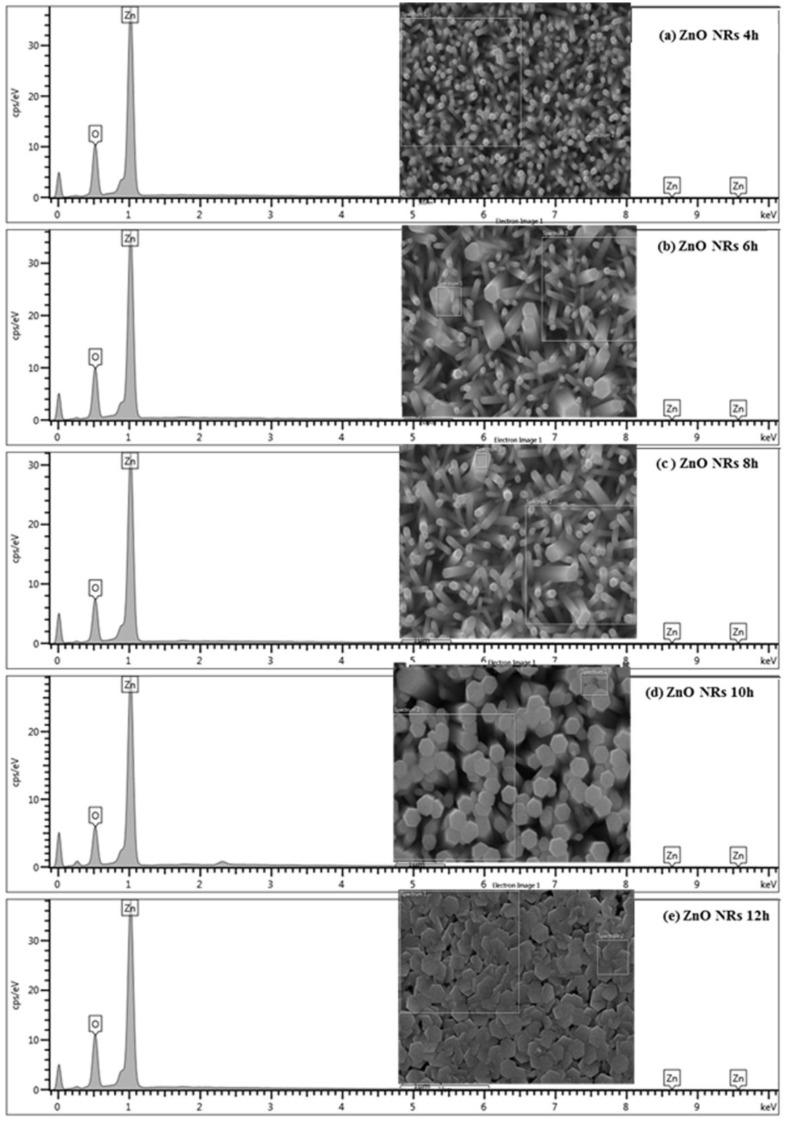
EDX spectra of ZNRAs prepared at various hydrothermal growth times with indicated scanned area in the FE-SEM image.

Figure 5 illustrates the EDX spectra of the ZNRAs obtained at different growth times which detected the chemical elements in the samples. Furthermore, the complete absence of other peaks in the ZNRAs connected to the defects/impurities indicated their high purity, supporting the XRD results. Table 1 shows the Atomic% of O and Zn in the ZNRAs obtained from EDX spectral analyses, which revealed the O:Zn ratio of approximately 1:1.27. In short, both FE-SEM images and EDX spectral analyses confirmed the successful production of stoichiometric ZNRAs at various growth times.

**Figure 6:**
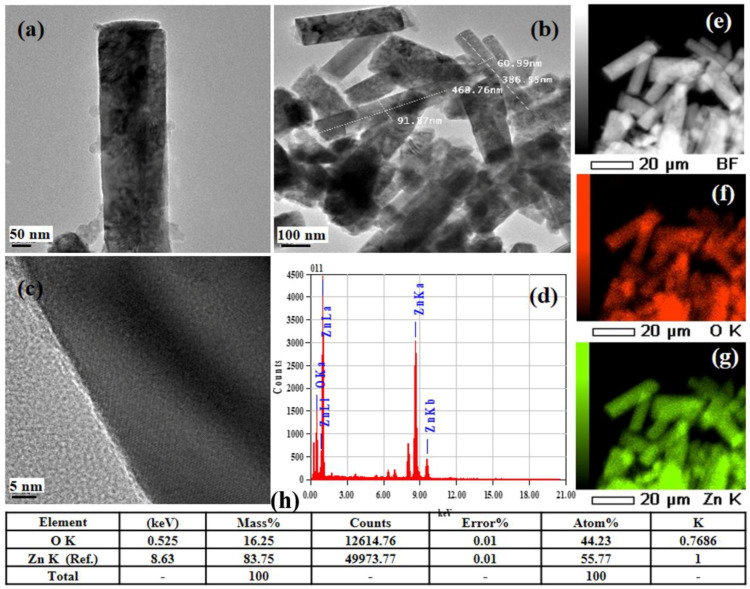
(**a**,**b**) TEM images, (**c**) HRTEM images, (**d**) EDX spectra of the ZNRAs-8h (Inset: EDX analyses for elemental composition), and (**e**–**h**) EDX maps.

Figure 6a–h depicts the TEM and HRTEM images, EDX spectra, and elemental maps together with their analyses of the ZNRAs produced at a growth time of 8 h. The TEM image (Figure 6a,b) of the sample demonstrates the rod-shaped ZnO nanocrystallite with mean lengths and diameters in the range of 300–500 nm and 40–90 nm, respectively. The HRTEM images of the sample (Figure 6c,d) indicated the nucleation and growth of ZNRAs, wherein the lattice fringe spacing of single ZNR was discerned to be 0.26 nm with growth lattice planer orientation of [002]. The EDX elemental maps (Figure 6e–g) revealed the presence of O and Zn in the sample. Again, the EDX spectra (Figure 6h) of the ZNRAs showed the prominent Zn and O peaks (without any impurities) in addition to the C peaks emerged from the instrument during measurement, verifying the right elemental composition of the sample. The estimated Atomic% (inset table) obtained from the EDX spectral analyses supported the FE-SEM results.

### 3.2. Effects of Growth Times on Optical Properties of ZNRAs

**Figure 7:**
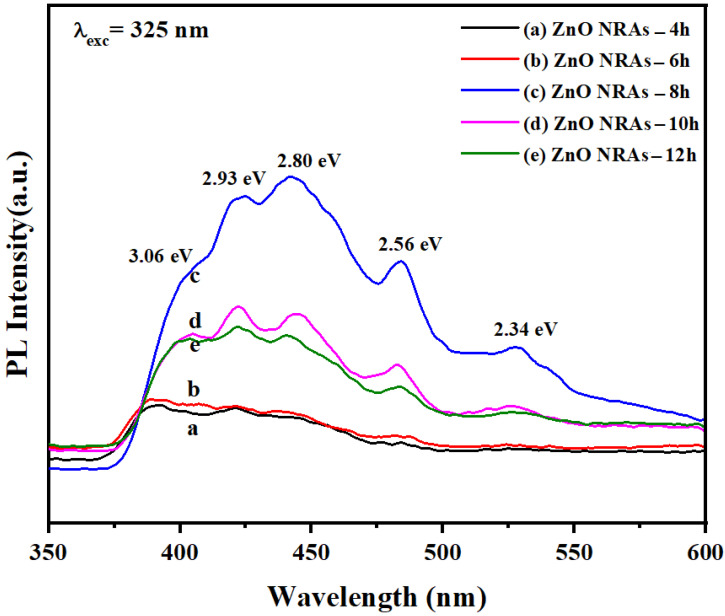
PL spectra of ZNRAs prepared at various hydrothermal growth times.

Figure 7 shows the hydrothermal growth times-dependent PL spectra of the ZNRAs obtained at 325 nm excitation comprising a near band edge UV emission peak and four deep levels visible emission peaks. These visible PL peaks were due to diverse intrinsic defects such as O and Zn vacancies, O and Zn interstitials, as well as O antisites present in the ZNRAs [36]. The observed emission peaks in the range of 394 to 403 nm (3.14–3.06 eV) correspond to the near band edge emission obtained from the defect states recombination across the conduction and valence state in ZNRAs [37,38]. The violet emission at 424 nm (2.93 eV) originated from neutral Zn interstitials (Zn_i_) shallow defects [37]. It is reported that the origin of this violet emission is due to the recombination of carriers (Zn_i_ and hole) in the valence state [39]. The blue peak centered at 441.9 nm (2.80 eV) was due to the singly ionized Zn vacancy (V_Zn_^−^) [40]. The blue-green peak positioned at 483.2 nm (2.56 eV) corresponded to the electronic transitions from the shallow donor levels of Zn_i_ to the acceptor levels of neutral V_Zn_ [41]. Additionally, the deep blue emission peak is due to the electronic transitions from the Zn_i_ to V_Zn_ levels due to their high symmetries [42]. The green PL peak at 527.6 nm (2.34 eV) was due to the radiative recombination of oxygen antisites across the conduction and valence states [41]. It was acknowledged that the green emission is due to recombining the trapped electrons in the doubly ionized O vacancies with the photo-generated holes [43].

The PL peak intensities increased with growth times from 4 to 8 h, and then, suddenly, decreased. Furthermore, the position of the band edge emission shoulders was red-shifted towards the lower energy value (from 394 nm to 406 nm) due to the increase in the length and diameter of ZNRAs at longer growth times. The observed reduction in the PL emission peak intensity of the ZNRAs grown at 10 and 12 h was due to decreased aspect ratios and nonradiative recombination of the photo-excited electrons [44]. It was asserted that our systematic procedure of ZNRAs growth could enable an effective transport of the photogenerated carriers (electrons and holes) and accumulation at the PEC electrodes surface through the O evolution reactions, leading to their efficient separation and carriers’ recombination inhibition. In brief, the ZNRAs formation can promote an effective separation of the photogenerated carriers and increase their lifetimes, thus achieving intense PL emission peaks, high photocurrent density, and photoconversion efficiency.

**Figure 8:**
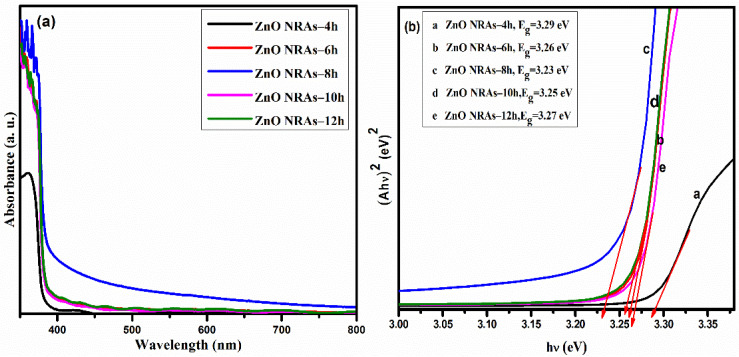
(**a**) The absorbance spectra and (**b**) Tauc plot (α*hv*)^2^ vs. energy (*hv*) of ZNRAs prepared at various hydrothermal growth times.

Figure 8a displays the growth times-dependent absorption spectra of the ZNRAs. The absorption edge of the ZNRAs showed the red-shift from 375.21 nm to 382.11 nm with the increase of corresponding hydrothermal growth times from 4 to 12 h. The samples grown at 4 and 6 h exhibited a prominent absorption in the near-UV region with the edge at 376 nm. The observed red-shift in the absorption edge and decreased in the absorbance of the ZNRAs beyond 8 h was mainly due to the increase in the mean diameters of the ZNRs. The saturation in the absorbance at growth times of 10 and 12 h was due to the saturation in the lengths and diameters of ZNRAs. However, the enhanced visible absorption at 8 h of growth time may be due to an improvement in the concentration of native defects (vacancies and interstitials) within the crystalline ZNRAs. Additionally, the random distribution of the ZNRAs grown at 8 h could strongly scatter the incoming photons, thus increasing their absorbance [45]. In short, the observed enhanced visible absorption and presence of native defects made the proposed ZNRAs (prepared at a growth time of 8 h) photoactive under visible light irradiation. Figure 8b shows the Tauc plot (α*hv*)^2^ vs. energy (*hv*) of ZNRAs prepared at various hydrothermal growth times. The intercept of the linear part of the plot on the energy axis provided the values of optical band gap energy (*E_g_*) of the ZNRAs.

**Figure 9:**
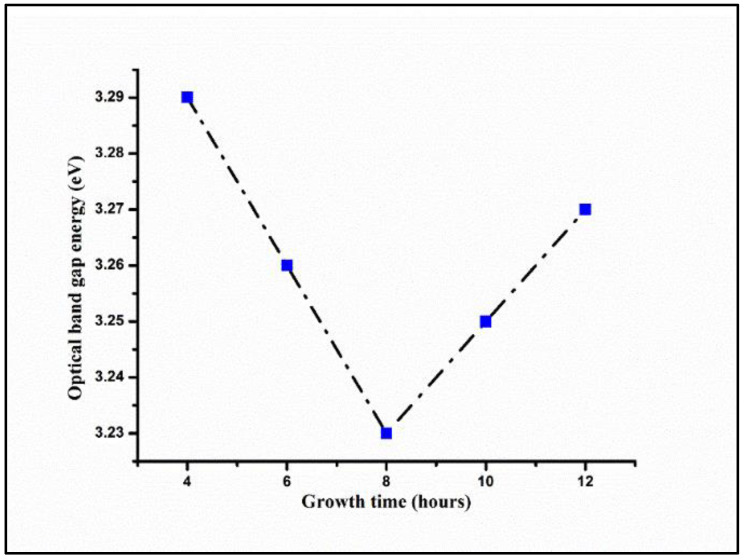
Hydrothermal growth times-dependent variation in the *E_g_* values of ZNRAs.

Figure 9 displays hydrothermal growth times-dependent variation in the *E_g_* values of ZNRAs. The values of *E_g_* of the ZNRAs were 3.39, 3.26, 3.23, 3.25, and 3.27 eV corresponding to growth times of 4, 6, 8, 10, and 12 h which were consistent with other findings [23,26,45]. The achieved decrease in the value of *E_g_* for the ZNRAs prepared at 8 h was mainly due to significant improvement in the nanocrystallinity. However, the obtained increase in the values of *E_g_* for the ZNRAs prepared at 10 h and 12 h was due the joining of adjacent crystalline planes of the NRs, leading to the formation of larger nanocrystalline rods (thicker with larger mean diameters and aspect ratios).

### 3.3. XPS Spectra of ZNRAs

**Figure 10:**
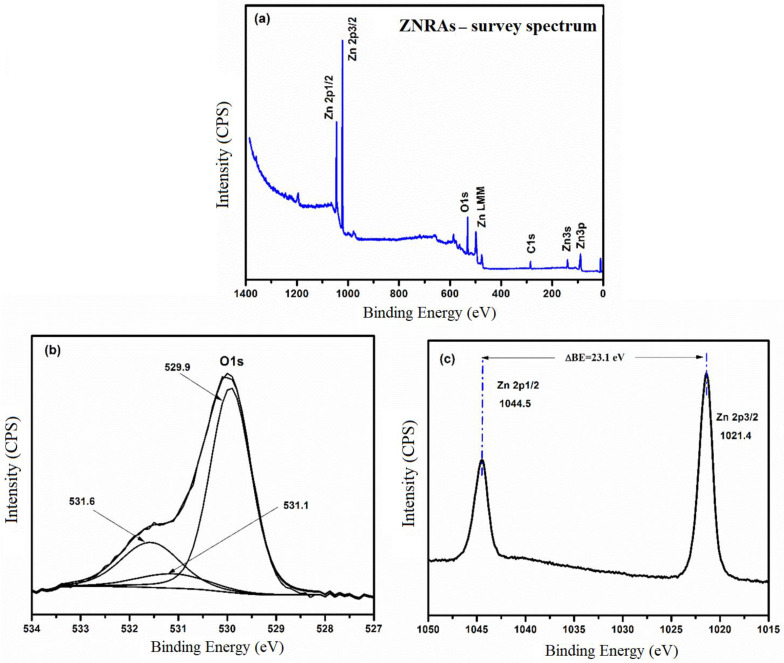
(**a**) Survey spectrum, (**b**) high resolution XPS spectrum of O 1s, and (**c**) Zn 2p of ZNRAs-8h.

Figure 10a illustrates the XPS spectrum of ZNRAs grown at 8 h which comprised of Zn, O, and C elements without any impurities. To standardize the binding energy peaks of all other elements present in the ZNRAs, C 1s peak at 284.6 eV was utilized as reference [46]. Figure 10b shows the O 1s spectrum fitted with three overlapping Gaussian peaks at 529.9, 531.1, and 531.6 eV. The peaks at 531.1 eV and 531.6 eV corresponded to the presence of O-H linkages and chemisorbed O such as weak bond of O_2_ passivated at the surface [47]. The peak at 529.9 eV was due to O^2−^ present in the wurtzite crystal structure of ZnO [48,49]. Figure 10c displays the spin orbit splitting of the 2p peak Zn of zinc wherein 2p_3/2_ of zinc occurred at 1021.4 eV and 2p_1/2_ of zinc appeared at 1044.5 eV with a binding energy difference (ΔBE) of approximately 23.1 eV, indicating the presence of Zn^2+^ in the ZNRAs. A comparison of the observed XPS spectral peaks of O and Zn with the other reported works [49,50] confirmed the existence of Zn^2+^ oxidation states of Zn in the proposed ZNRAs. The ΔBE value of Zn 2p peak without chemisorbed H_2_O on the ZNRAs surface was shown to be 23.1 eV [51].

### 3.4. Photoelectrochemical Performance of the ZNRAs

**Figure 11:**
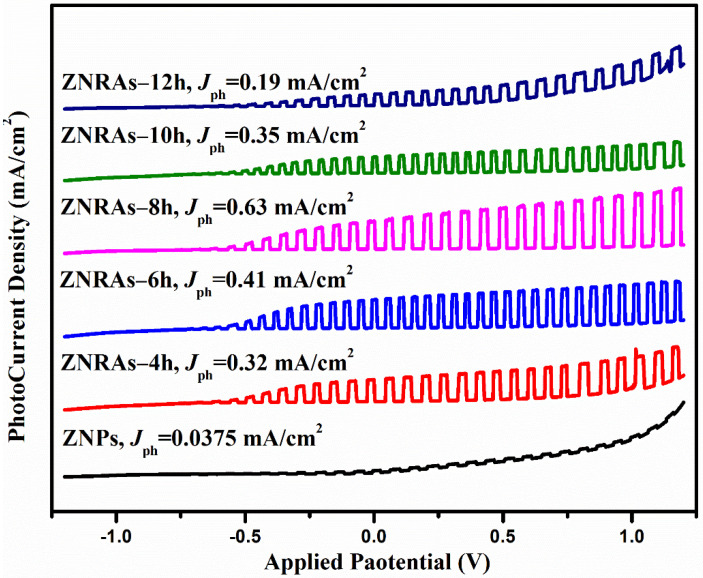
LSV response of ZNPs (seed layer) and ZNRAs prepared at various growth times.

Figure 11 shows the LSV plot of the PEC designed with photoanodes made from ZNPs and ZNRAs obtained at various hydrothermal growth times. The PEC performance was continuously enhanced with the increase of the growth times from 4 to 8 h and then dropped at 10 and 12 h. The observed reduction in the PEC performance with the increase of growth times beyond 8 h may be due to the hydrolyses of OH^-^ in the water solution from HMTA [9,52]. Because the growth solution is not refreshed during the hydrothermal process, thus, the quantity of precursor ions was limited in the closed system wherein the growth process inhibition of the ZNRAs due to prolong growth might have required more Zn^2+^. Consequently, the photocurrent density was affected due to the decrease in the aspect ratio. These results were consistent with other findings [11,21,52]. The ZNRAs grown at 4, 6, 8, 10, and 12 h produced the *J_ph_* values of 0.32, 0.41, 0.63, 0.35, and 0.19 mA/cm^2^, respectively, indicating the strong dependence of photocurrent density on the hydrothermal growth durations of ZNRAs. The observed improvement in the photocurrent density with the increment of the growth times is directly correlated to the increase of the crystalline ZNRs diameters as shown in FE-SEM images Figure 4, leading to enhanced light harvesting and efficient absorption. In addition, the transport of the induced photoelectron and effective diffusion transfer of the holes within the electrolyte to the redox species S^2−^ and SO_3_^2−^ from the electrolyte that acted as holes scavenger was facilitated, leading to a significant reduction in the nonradiative recombination losses [48].

**Figure 12:**
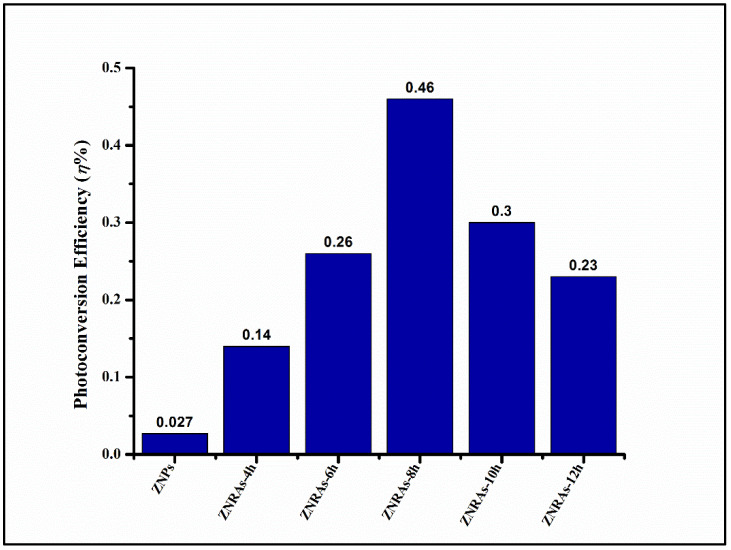
Values of *η*% for ZNPs and ZNRAs obtained at various hydrothermal growth times.

Figure 12 displays the photoconversion efficiency (*η*%) of the ZNPs and ZNRAs obtained at various hydrothermal growth times. The obtained reduction in the *J_ph_* value of ZNRAs grown for 10 h and 12 h may be due to the decrease in the effective surface areas and increase in the optical band gap energies of ZNRAs. The ZNRAs grown for 8 h showed the optimum photocurrent density of 0.63 mA/cm^2^ and photoconversion efficiency of 0.46%, indicating that the PEC photoanode made using this sample could offer an effective transport pathway of the photogenerated carriers and significant reduction in the electron diffusion distance. It was asserted that the production of ZNRAs with large aspect ratios are key factors to achieve high photoconversion efficiency and photocurrent density of PEC with such ZNRAs as the photoanode, supporting the FE-SEM and optical absorption results.

Table 2 compares the photoelectrochemical water-splitting cell performance of the proposed ZNRAs as the photoanode with other state-of-the-art works reported in the literature. The distinct physiochemical and optical attributes of the proposed ZNRAs produced by the systematic sol-gel spin-coating combined hydrothermal technique enabled them to achieve their outperforming traits compared to others. The grown ZNRAs showed high photoconversion efficacy, optical sensitivity, photocatalytic activity, mechanical strength, as well as thermal and electrical conductivity beneficial to PEC electrode applications. It was concluded that our proposed approach might constitute a basis for the production of ZNRAs at a large scale, which is desirable for practical applications.

## 4. Conclusions

The proposed ZNRAs were established to be advantageous for developing high-performance PEC photoanodes. A series of ZNRAs was deposited on ITO substrates at varying hydrothermal growth times using a two-step process (sol-gel spin coating unified hydrothermal technique). The variation of growth times significantly influences the micro-structural, morphological, optical, and photo-electrochemical properties of the ZNRAs. The obtained ZNRAs demonstrated wide surface areas, excellent light harvesting traits, and superior carrier transport compared to ZNPs. The ZNRAs grown at 8 h exhibited an optimum PEC performance in photocurrent density (0.63 mA cm^−2^) and photoconversion efficiency (0.46%), which is 17 times higher than ZNPs.

## Figures and Tables

**Table 1:** Atomic% of Zn and O in the ZNRAs obtained from EDX spectral analyses.

Element	ZNRAs-4h	ZNRAs-6h	ZNRAs-8h	ZNRAs-10h	ZNRAs-12h
O	45.76	45.51	41.97	40.09	46.86
Zn	54.24	54.49	58.03	59.91	53.14
Total:	100	100	100	100	100

**Table 2:** Photoelectrochemical water-splitting cell performance of the proposed optimum ZNRAs-8h as the photoanode when compared with other reports in the literature.

Photoelectrode	Growth Method	Electrolyte	Illumination Intensity	*J*_ph_ (mAcm^−2^)	Ref.
ZNRs	Hydrothermal	0.1 M Na_2_S + 0.1 M Na_2_SO_3_	100 mW/cm^2^	0.37 at 0.0 V vs. Ag/AgCl	[23]
ZNRs	Hydrothermal	0.1 M Na_2_S + 0.1 M Na_2_SO_3_	100 mW/cm^2^	0.337 at 0.5 V vs. Ag/AgCl	[26]
ZnO (nanotetrapod)	Thermal evaporation	0.5 M Na_2_SO_4_	100 mW/cm^2^	0.40 at 0.8 V vs. Ag/AgCl	[53]
ZNRs	Hydrothermal	0.1 M Na_2_S + 0.1 M Na_2_SO_3_	100 mW/cm^2^	0.194 at 0.5 V vs. Ag/AgCl	[20]
ZnO (nanocoral)	RF magnetron, thermal oxidation	0.5 M Na_2_SO_4_	125 mW/cm^2^	0.25 at 1.2 V vs. Ag/AgCl	[54]
ZNRs	Hydrothermal	0.5 M Na_2_SO_4_	100 mW/cm^2^	0.62 at 1.2 V vs. RHE	[48]
ZnO (nanotubes)	Electrochemical anodization	0.5 M Na_2_SO_4_	100 mW/cm^2^	0.52 at 0.25 V vs. SCE	[55]
ZNRs	Vacuum annealed	0.5 M Na_2_SO_4_	100 mW/cm^2^	0.60 at 1.23 V vs. RHE	[56]
ZNRAs-8h	Hydrothermal	0.1 M Na_2_S + 0.1 M Na_2_SO_3_	100 mW/cm^2^	0.63 at 0.5 V vs. Ag/AgCl	Current study

## Data Availability

Not applicable.
